# Understanding end-user contexts and identifying design preferences of an artificial intelligence-based clinical decision support tool for early autism detection

**DOI:** 10.1093/jamiaopen/ooag145

**Published:** 2026-07-23

**Authors:** Adesuwa Emovon, Lauren Driggers-Jones, Matthew Engelhard, Gary Maslow, Geraldine Dawson, Benjamin A Goldstein, Lauren Franz

**Affiliations:** College of Medicine, Penn State, Hershey, PA, 17033, United States; Department of Psychiatry & Behavioral Sciences, Duke University School of Medicine, Durham, NC, 27710, United States; Department of Biostatistics and Bioinformatics, Duke University School of Medicine, Durham, NC, 27710, United States; Department of Psychiatry & Behavioral Sciences, Duke University School of Medicine, Durham, NC, 27710, United States; Department of Pediatrics, Duke University School of Medicine, Durham, NC, 27710, United States; Department of Psychiatry & Behavioral Sciences, Duke University School of Medicine, Durham, NC, 27710, United States; Department of Pediatrics, Duke University School of Medicine, Durham, NC, 27710, United States; Department of Biostatistics and Bioinformatics, Duke University School of Medicine, Durham, NC, 27710, United States; Department of Pediatrics, Duke University School of Medicine, Durham, NC, 27710, United States; Department of Psychiatry & Behavioral Sciences, Duke University School of Medicine, Durham, NC, 27710, United States

**Keywords:** autism, clinical decision support, user-centered design, electronic health records, artificial intelligence

## Abstract

**Objectives:**

Building on innovations for autism detection—where artificial intelligence (AI)-based models monitor clinical data within electronic health records—this study evaluates the context for clinical decision support (CDS) deployment and identifies design preferences.

**Materials and Methods:**

This observational study utilized contextual inquiry to elicit perspectives from 8 clinicians and twenty caregivers during 18- to 24-month well-child visits at Duke-affiliated clinics. Data were analyzed using rapid qualitative analysis techniques.

**Results:**

Workflow analysis identified 6 user tasks, 3 technology-user interactions, and 5 clinical decision points. Technologies that streamlined screening included patient portals, digital tablets, and note templates. Clinicians identified 2 major barriers—limited screening tool accuracy and challenges in implementing follow-up steps—and 3 facilitators: electronic screening, early intervention provider input, and staff referral coordination support. For design, CDS should include clear, actionable outputs, with explanations of prediction data, visual summaries linked to next steps, and educational resources. Embedding CDS within the EHR, with outputs delivered at key points during the clinical encounter, along with caregiver-facing materials, would improve workflow efficiency.

**Discussion:**

Findings highlight key integration points for an autism detection AI-based CDS tool and stress the need for clinical utility and caregiver-centered communication. Effective design requires alignment with clinical workflow, including the timing of outputs, meaningful explanations, and integration with caregiver communication.

**Conclusion:**

Findings will inform the design of an AI-based CDS tool for autism detection, providing workflow-informed integration points and user preferences. Future work should refine explainability and optimize delivery of outputs within clinical encounters to support decision-making and caregiver engagement.

## Background and significance

Autism is a neurodevelopmental condition associated with differences in social and communication abilities and with restrictive and repetitive behaviors.^1^ The estimated autism prevalence in the United States is 1 in 31 among individuals aged 8 years,^2^ with approximate annual costs of over US$461 billion associated with supports and services and decreased economic participation.^3^ Early identification of autism and intervention are key to improving long-term outcomes and reducing the need for services at a later age.^4^ However, early detection remains a significant challenge.^5^^,^^6^ While autism can be accurately identified by 24 months, the average diagnostic age in the United States is 47 months.^2^^,^^7^ Regional variation in diagnostic age is notable: California has the earliest average age at 36 months, whereas Laredo, Texas has the latest average age at 70 months—differences that likely stem from service access and availability.^2^

To improve equitable early detection of autism, the American Academy of Pediatrics (AAP) endorses universal autism screening during 18- and 24-month well-child visits with the Modified Checklist for Autism in Toddlers, Revised with Follow-Up (M-CHAT-R/F), a caregiver-completed screening tool.^5^ While universal screening is useful, mounting evidence suggests that the accuracy of caregiver-completed tools may be impacted by caregiver factors, including literacy, language, and education, and clinician factors, including training and experience, administration procedures, and implicit biases.^8–11^ Furthermore, in general pediatric populations, the M-CHAT-R/F has been found to over-identify children with other developmental concerns, and under-identify those with autism, as about a quarter of children who screen negative later receive an autism diagnosis.^12^ These findings highlight the need to develop and implement new methods to ensure that a greater proportion of children can benefit from early detection and intervention.

Recent innovations in enhance early detection of autism include the development of artificial intelligence (AI)-based models that monitor routine clinical data within electronic health records (EHR).^13–15^ These AI-based models analyze a wide range of clinical information—such as diagnostic codes, vital signs, and medical procedures—to generate predictive insights. Studies have found that by 30 days of life, AI-based models using EHR data can achieve an area under the receiver operating characteristic curve of 0.76, which is comparable to that of screening tools such as the M-CHAT-R/F completed at 18- and 24-month well-child visits.^13^ The performance of such AI-based models for early detection of autism improves with age, especially when incorporating insights from clinical notes through natural language processing, and reaches a receiver operating characteristic curve of 0.80 by one and a half years.^14^^,^^15^ An appeal of these AI-based models is that they do not require additional input from caregivers to make autism predictions, but can be used to complement standard care to make screening more consistent, objective, and effective.

Such clinical decision support (CDS) tools—which are digital systems that can be integrated into EHR—can provide timely, personalized information to clinicians and families at the point of care to support shared decision-making.^16^ To our knowledge, current autism CDS tools do not incorporate AI-based predictive models, but instead focus on automating aspects of caregiver-completed screening within EHR, including screening reminders, automated scoring of screening tools such as the M-CHAT(R/F), and support for referral workflows, with some systems further enhancing integration by auto-populating results into clinical note templates and embedding referral pathways.^17–21^ Among these, the most comprehensive approach is exemplified by the Child Health Improvement Through Computer Automation clinical decision support system, a rules-based platform that prompts caregivers to report autism-related concerns during recommended well-child visits, in alignment with AAP guidelines, facilitates electronic screening and scoring, tracks referral and diagnostic outcomes longitudinally, and provides families with navigation resources.^20^

As such, these systems are primarily designed to improve adherence to recommended screening practices rather than to generate individualized predictions of autism likelihood or support clinical decision-making based on longitudinal EHR data. This distinction highlights a key gap in the current CDS landscape, as predication-based systems introduce additional considerations, including explainability, trust, and integration with caregiver-facing communication, that are not addressed by existing tools. Although AI-based CDS tools have the potential to improve care, most prediction models have not been not adopted into clinical practice.^22^^,^^23^ Implementation barriers that hinder the usability and trustworthiness of AI-based CDS tools include challenges integrating these systems within the EHR, clinical workflow disruptions, and concerns related to tool explainability, generalizability and real-world impact.^24–27^

To develop an AI-based CDS tool for early autism detection that can be used effectively in routine clinical practice, it is critical to understand the implementation context—such as workflows including the technology interface (eg, EHR systems) utilized during well-child visits—and to engage end-users in the design process.^28–31^ The use of best practice frameworks in the areas of CDS design, implementation science and human-centered design can support the development of user-friendly systems aligned with clinician workflow and decrease implementation barriers.^28–31^ In terms of CDS design, the Five Rights framework outlines key considerations for successful workflow integration of CDS tools, informing design and functionality by guiding how information is structured and presented to the end-user.^29^^,^^32^ The Five Rights framework includes communicating the “right information, to the right person, in the right format, through the right channel, and at the right time” through established clinical pathways.^26^ In terms of implementation science, the Consolidated Framework for Implementation Research (CFIR) supports identification of multilevel contextual factors that influence CDS tool adoption.^30^^,^^31^ Specifically, CFIR can guide CDS tool complexity, delineate the clinical environment, identify external influences, and explore beliefs, knowledge, and attitudes of individuals who will interact with the tool. Finally, Maguire’s Framework on human-centered design supports the development of systems that are more intuitive and satisfying to use by focusing on end-user needs.^28^ Maguire’s Framework guides understanding of clinician workflow and decision-making, operational environments, and integration within the EHR.

## Objective

Building on our team’s work to enhance early autism detection using AI-based models that monitor routine EHR data,^13–15^ this manuscript describes a first step toward understanding the context for deployment of a CDS system that provides individualized screening and referral recommendations at the point of care. Using an approach informed by best practice frameworks in CDS design, implementation science and human-centered design the objective of this study was to: (1) evaluate end-user contexts and (2) identify design and content preferences to inform prototype development. To address challenges that other CDS tools have faced that limit clinical integration, in this first phase gathered the perspectives of end-users—clinicians and caregivers.

## Materials and methods

### Study design

This observational study utilized a semi-structured field note template to guide direct observations of 18- to 24-month well-child visits and an interview guide to elicit clinician and caregiver perspectives.

### Setting and participants

The study took place within the Duke University Health System (DUHS), a mid-sized health system that consists of 3 hospitals and over 150 affiliated clinics, which provide primary care for approximately 85% of children in Durham County, North Carolina.^33^ Participants included clinicians and caregivers of children who attended 18- to 24-month well-child visits at DUHS-affiliated clinics. Clinician inclusion criteria included: (1) working with pediatric populations and being employed by DUHS, (2) conducting autism screening as part of regular practice, (3) working with children from birth through age 6 years, and (4) being willing and able to participate in study activities. Caregiver inclusion criteria included: (1) being 18 years or older, (2) being the caregiver of a child who attended an 18- to 24-month well-child visit within DUHS, and (3) being willing and able to participate in study activities.

### Measures

The demographic questionnaire included 4 questions that collected clinician age, gender, ethnicity, and race, consistent with National Institutes of Health reporting standards. The Contextual Inquiry included a field note template and interview guide developed by the research team that drew on constructs from the Five Rights of CDS, CFIR and Maguire’s Framework.^28^^,^^30^^,^^31^^,^^34^^,^^35^ The Contextual Inquiry was designed to elicit insights into: (1) clinical workflow during 18- to 24-month well-child visits, including how and when autism screening occurred and the resulting clinician actions, (2) technology-user interactions, including technology that supported or hindered clinician actions, and (3) CDS content and design preferences. The field note template guided observations during well-child visits, followed by individual interviews with clinicians and caregivers. Clinician interview guides included visual examples of CDS tools to elicit feedback on alert design and format preferences. The Contextual Inquiry and Caregiver Interview are included in Supplementary Methods.

### Procedures

Study procedures received approval from the Duke Health Institutional Review Board (Pro00111371) and the Duke Primary Care Research Consortium. Duke-affiliated clinics were identified through discussions with the Primary Care Research Consortium. Once identified, clinic leads were approached via email to gauge their willingness to participate. If interested, clinic lead provided the study team with clinician contact information, who were approached via email. Once a participant indicated interest in participating, they were emailed an eConsent form. Study staff reviewed each consent to confirm completion before proceeding. For caregivers observed during clinic visits, the clinician was provided with a brief IRB-approved consenting script to review with the caregiver. After reviewing the script, the clinician confirmed consent, and a study staff member entered to observe the visit.

In the first phase of the contextual inquiry, clinicians were observed by study staff during 18- to 24-month well-child visits. The field note template was used to document clinician workflow, including the technology-user interactions supporting autism screening and clinical decision points related to this process. A total of 17 contextual inquiry sessions were conducted, each lasting 25-30 minutes (total observation time approximately 8 hours). In the second phase of the contextual inquiry, study staff conducted interviews with clinicians to further detail the clinical workflow, technology-user interactions, and barriers and facilitators to existing developmental screening and referral tools. Interviews with clinicians and caregivers also elicited feedback on the design and content preferences for an AI-based CDS tool for autism detection, including navigating next steps following identification of developmental concerns. Clinicians completed the interview after 2 or 3 18- to 24-month well-child visits had been observed by study staff. Caregivers completed the interview immediately following their well-child visit. Caregiver interviews lasted ∼10 minutes, while clinician interviews lasted ∼20 minutes and were conducted after completion of observations. Study staff entered data into the field note survey instrument and interview template in REDCap.^36^

Members of the study team had prior experience working with the Duke University Health System and in autism-related clinical research settings. This familiarity supported interpretation of clinical workflows but may have influenced data collection and analysis. To mitigate potential bias, structured field note templates were used, and findings were reviewed collaboratively across the research team to promote consistency.

### Data analysis

Rapid qualitative analysis techniques were employed.^37^^,^^38^ Following each clinician’s observed well-child visit, study staff who led data collection and data analysis debriefed to ensure comprehensive documentation.^39^ Data analysis was led by one team member, who condensed each contextual inquiry into template summaries that organized key findings into 6 pre-specified constructs informed by the Five Rights, CFIR, and Maguire’s Framework including: user tasks, technology-user interactions, clinical decision points, attitudes toward current developmental screening, and CDS content and design preferences.^28–31^ Field notes and interview data were jointly synthesized within each template summary. Template summaries were reviewed during team meetings to establish consistency in the coding approach and discuss coding questions. Data from template summaries were then integrated into a matrix to summarize and compare findings across contextual inquires and identify broader themes.^37^^,^^38^ The workflow diagram was developed using a systematic visual mapping approach—employing Lucidchart and standardized process mapping symbols—to document user tasks, technology-user interactions, and clinical decision points, with the goal of identifying opportunities for integrating AI-based CDS tools for autism detection within the clinical workflow.^40–42^

## Results

Participants were drawn from 3 DUHS-affiliated clinics—2 pediatric practices and 1 family medical practice—and consisted of 8 clinicians (6 pediatricians and 2 family medicine physicians) along with twenty caregivers whose children attended 18- to 24-month well-child visits. Clinicians’ ages ranged from 35 to 53 years. Most were female (*n* = 6), White (*n* = 6), and Non-Hispanic/Latino (*n* = 8).

### Clinical workflow for autism identification during 18- to 24-month visits

**Figure 1.**
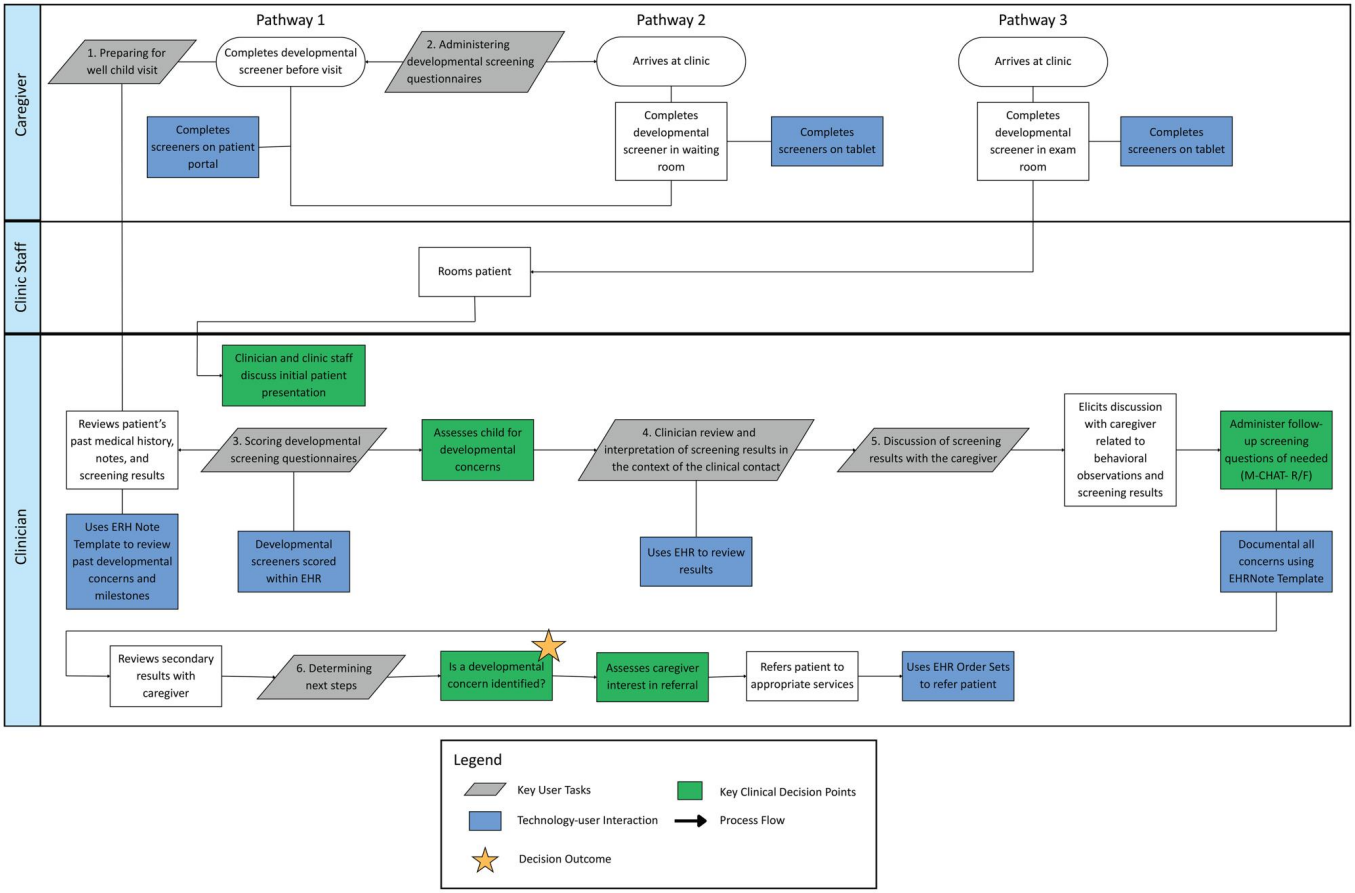
Workflow diagram for 18- to 24-month well-child visits.

Figure 1 illustrates a workflow diagram summarizing user tasks, technology-user interactions, and clinical decision points related to autism screening during the 18- to 24-month well-child visit.

#### User tasks

Six key user tasks—specific activities necessary to complete a well-child visit related to autism screening^31^—that involved caregivers, clinic staff, and clinicians were identified. The first task was well-child visit preparation. As shown in Pathway 1, prior to clinic arrival, some caregivers completed screening questionnaires and documented concerns about their child’s development—either independently or through discussion with their partner. Before seeing the patient, clinicians reviewed available screening results and other relevant EHR-based information. The second task involved administration of screening questionnaires. Caregivers completed questionnaires before the well-child visit (Pathway 1), in the waiting room with assistance from clinic staff (Pathway 2), or in the exam room prior to clinician arrival (Pathway 3). The third task was scoring of screening questionnaires, a process that was automated. The fourth task included clinician review and interpretation of screening results. This occurred either before entering the patient room or during the visit, depending on when questionnaires were completed. The fifth task included discussion of screening results. When developmental screening indicated potential concerns—or when caregivers raised questions—clinicians consistently reviewed the results. The sixth task included decisions on next steps. When concerns were identified, clinicians took various actions, such as administering follow-up screening, recommending ongoing monitoring, or initiating a referral for further assessment and/or early intervention.

#### Technology-user interactions

Three technology-user interactions—defined as technologies that supported user tasks, related to screening^31^—utilized by caregivers, clinic staff, and clinicians were identified. The first involved patient portals—secure, caregiver-facing digital dashboards integrated within the EHR. These portals enabled caregivers to complete screening prior to well-child visits, supporting visit preparation. The second involved digital tablets, which were employed by clinic staff to administer the M-CHAT-R/F and the Survey of Well-being of Young Children (SWYC).^11^^,^^43^ The third centered on EHR note templates—embedded within the EHR and customized to the visit type. Note templates supported documentation of developmental concerns and relevant history, with auto-population of screening results—including scores, score interpretation, follow-up recommendations, and order sets to streamline referrals and enhance clinical decision-making.

#### Clinical decision points

Five clinical decision points—defined as points of clinical judgment within the workflow that shape subsequent actions supporting developmental screening, including decisions about further evaluation, referral, documentation, and communication with caregivers^31^—were identified. The first decision point was to obtain input related to child developmental concerns from clinic staff who brought the child and caregiver to the exam room and performed intake tasks. This reflects a clinician-directed decision about what information is needed to guide subsequent assessment. Staff questions were informed by clinician review of screening results. The second decision point was to assess the child for developmental concerns. Review of screening results, input from clinic staff, and caregiver reports of developmental concerns supported a targeted clinical developmental assessment early in the encounter, which for autism focused on behaviors such as eye contact, smiling, babbling, and other forms of expressive communication. The third decision point was to administer follow-up screening. When autism was a concern, the follow-up interview for the M-CHAT-R/F was administered to clarify caregiver responses to items on the M-CHAT. The fourth decision point was caregiver communication of developmental concerns, which involved clinician judgment regarding how, when, and what information to share, with explanations focusing on observed behaviors. The fifth decision point was assessment of caregiver interest in referral for further developmental evaluation and/or early intervention, where clinicians explored caregiver openness to and desire for these suggested next steps.

### Preferences for AI-based autism detection CDS tool

#### Perceptions of current autism screening approach

Clinicians identified barriers and facilitators of the current autism screening approach. Two main barriers were identified. The first related to the accuracy of screening tools, which clinicians reported overestimated child risk for developmental delays, leading to unnecessary caregiver concern. In addition, clinicians noted that screening questions often did not align with caregiver-identified developmental concerns and were sometimes misunderstood. Furthermore, clinicians noted that accurately interpreting the M-CHAT-R/F was challenging when it was administered in languages other than English. The second barrier related to implementing next steps after a positive screen. Clinicians noted that discussion of screening results was often lengthy, which presented challenges due to time constraints of well-child visits. Additionally, clinicians reported delays along the referral pathways due to shortages of qualified providers to conduct developmental evaluations, and inconsistent caregiver engagement with follow-up recommendations. Three facilitators were identified. The first related to electronic administration of screening tools—through patient portals or tablets with automated scoring—which helped reduce administrative burden. Particularly when completed prior to well-child visits, this approach facilitated clinician preparation and interpretation of results. The second facilitator involved input from early intervention providers—such as occupational or speech therapists—who offered additional assessment support and recommendations, thereby enhancing clinical decision-making. The third facilitator included staff support—such as patient navigators for referral coordination of evaluations and early intervention—which made next steps following a positive screen more efficient and increasing adherence to recommended care.

#### Content and design preferences of AI-based CDS tool

Overall, clinicians wanted an AI-based CDS tool for autism detection that enhanced current developmental screening and supported timely connection of families to services. Table 1 summarizes clinician and caregiver content and design preferences aligned with the Five Rights framework.^32^ In terms of “right information,” clinicians wanted a breakdown of the specific EHR data that informed autism likelihood predictions, along with an explanation of how the AI-based system worked. Clinicians also wanted actionable guidance on next steps following a positive screen. Similarly, caregivers wanted to understand which specific EHR data contributed to autism likelihood predictions, and the role of AI in the detection process, noting this information may impact their trust in the autism detection system. Caregivers also wanted summaries of screening results and educational resources outlining developmental milestones, follow-up timelines, and actionable guidance on next steps, including a list of local referrals. In terms of “right person,” to support shared decision-making, the CDS tool should be targeted to clinicians during routine clinical interactions to guide conversations and enhance clinical judgment. Caregivers expressed the need for direct clinician communication during the well-child visit—supplemented by educational resources—focused on the developmental concerns that were identified and the potential impact on their child. In terms of “right format,” clinicians indicated a preference for summary scores combined with visual elements, such as graphs featuring color-coded stratification of autism likelihood linked to specific actions to support decision-making and that could also be easily reviewed with caregivers. For example, for children at higher likelihood of autism, the AI-based CDS tool would provide a likelihood score alongside likelihood stratification in a color that would draw attention to the urgency of additional clinician actions related to follow-up screening and referrals. In addition, clinicians requested “scripts” to reference during conversations with caregivers that explained how the AI-based CDS system worked. Furthermore, clinicians noted that the tool should include a progress tracking feature to monitor when evaluations were scheduled and completed. Caregivers wanted clear, digestible summaries of results, educational materials, and local resources. In terms of “right channel,” the AI-based CDS tool should be incorporated into the EHR-platform, with interruptive alerts that pause the workflow and redirect clinician attention to screening information. Caregiver-facing materials should be delivered via clinicians and the supported by paper or electronic informational handouts, based on caregiver preference. Finally, in terms of “right time,” clinicians preferred information to be available prior to the visit when the EHR was opened during chart review, with caregiver-facing information summaries available at the end of the well-child visit. Alerts should be timed to avoid workflow disruption unless they are critical and provide useful, actionable information—otherwise, additional alerts would add to the burden of the existing system. To support flexibility, clinicians recommended that the CDS tool include an acknowledgment option allowing them to defer interaction until later. However, the timing of alert acknowledgment should be tailored to the length of the well-child visit. Caregivers wanted AI-based screening results to be communicated during the well-child visit, to allow for discussion and questions with the clinician.

**Table 1.** Content and design preferences of AI-based CDS tool.

CDS right	Clinician preferences	Caregiver preferences
Right information 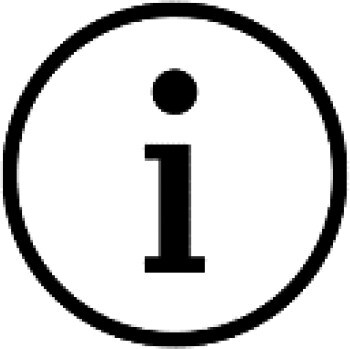	Breakdown of EHR data informing predictions Explanation of how the AI-based system works Actionable next step guidance following a positive screen	Understanding which EHR data contributed to predictions Role of AI in detection process Summaries of screening results Educational resources and milestone guidance Local referral information
Right person 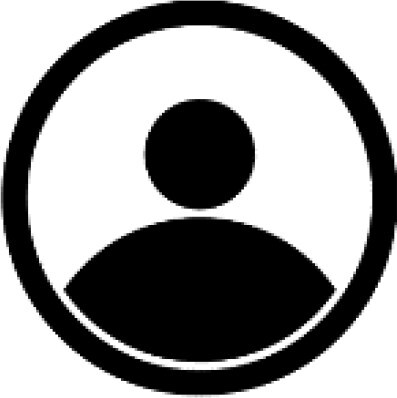	Targeted to clinicians during well-child visits to support decision-making	Direct clinician communication Supplemented by educational resources explaining developmental concerns and impact
Right format 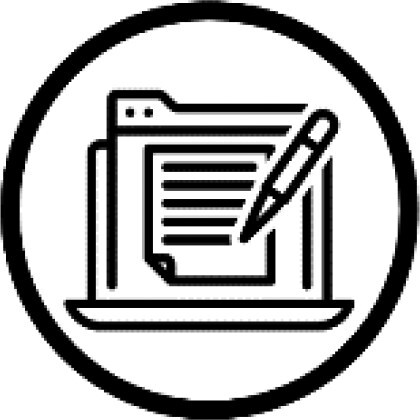	Summary scores with visual elements linked to actions (eg, color-coded graphs) Scripts to support caregiver conversations Progress tracking for next steps	Clear, digestible summaries of results, educational materials and local resource lists
Right channel 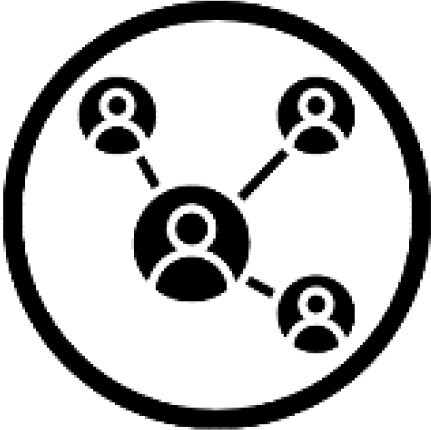	Embedded in EHR with interruptive alerts that redirect attention to screening info	Delivered via clinician communication Supported by paper or electronic handouts based on preference
Right time 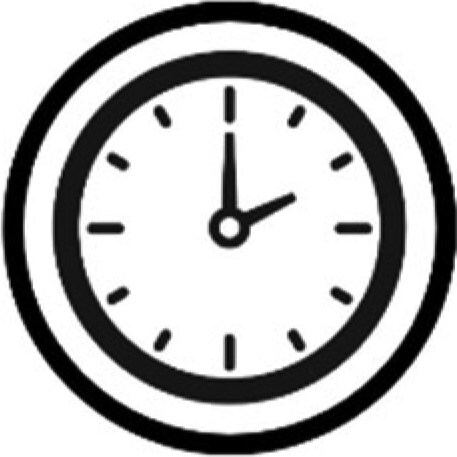	Information available during chart review before visit Caregiver-facing summaries at end of visit Alerts only when actionable Acknowledgment option to defer interaction Timing of deferment tailored to visit length	Shared during well-child visit for clinician discussion and questions

## Discussion

This study aimed to assess end-user contexts and uncover design and content preferences to support the development of an AI-based CDS tool for early autism detection. This work builds on existing autism CDS approaches, which primarily automate screening and referral workflows,^17–21^ by identifying how AI-based, prediction-driven CDS tools can be integrated into real-world clinical care. During 18- to 24-month well-child visits, we identified 6 user tasks, 3 technology-user interactions, and 5 clinical decision points. These findings map how autism screening unfolds in practice and identify specific points where CDS could support decision-making without disrupting workflow. Clinicians highlighted limitations of current screening approaches, particularly related to accuracy and follow-up implementation, alongside facilitators such as electronic screening and staff-supported referral coordination. Across participants, there was consistent emphasis on actionable outputs, including visual summaries linked to next steps, caregiver-facing materials, and clear connections between data and recommendations. Embedding the tool in the EHR with well-timed alerts and caregiver-facing resources during the visit was seen as key to improving workflow efficiency and follow-up adherence.

Overall, our findings identify where an AI-based CDS tool for autism detection could be integrated within the clinical workflow, and highlight that successful implementation will require a dual focus on clinical utility and caregiver-centered communication, supported by actionable guidance, transparency, and seamless integration into existing processes. These findings extend existing CDS principles by specifying how timing, content, and workflow alignment operate in the context of autism screening.

Importantly, participants provided specific guidance on what constitutes the “right time” and “right content” for CDS in this setting. Clinicians indicated that information should be available during chart review prior to the visit, while CDS outputs should be surfaced during the visit to inform assessment and caregiver discussion. Caregivers emphasized that results should be shared during the well-child visit to allow for real-time discussion and questions. Together, these findings suggest that CDS should function as an integrated support across multiple stages of the visit, rather than as a single alert.

Alert design was closely tied to these workflow considerations. Clinicians emphasized that alerts should be delivered only when actionable and should avoid disrupting workflow unless clinically urgent. Prior work shows that increasing alerts, particularly repeated or low-value alerts, reduces clinician engagement.^44^ In this context, threshold-based triggering (eg, surfacing alerts only when prediction confidence exceeds a defined level) may help prioritize high-value signals. Clinicians also recommended the inclusion of an acknowledgment option to allow deferral of interaction, with timing tailored to the length of the well-child visit.

These findings also highlight how alert timing may directly influence trust and sustained use of CDS. Prior work suggests that clinician engagement with CDS depends not only on alert frequency, but also on perceived relevance and timing within the clinical workflow, with poorly targeted or poorly timed alerts more likely to be dismissed.^45^ One potential design approach is to reserve interruptive alerts for high-confidence predictions, while presenting lower-confidence information in non-interruptive formats (eg, within chart review), thereby supporting clinical awareness without adding unnecessary disruption. This approach aligns with CDS principles emphasizing relevance, efficiency, and workflow integration.^46^

While explainability of an AI-based CDS tool for autism detection—such as the specific EHR inputs used to generate predictions—was identified as an important factor supporting use of the system, there is lack of consensus on optimal levels of model explainability, with concerns that model explanation may be inconsistent or misleading, thereby potentially compromising clinical decision-making.^47–49^ In this study, clinicians emphasized the need for concise, clinically meaningful explanations, such as brief summaries of contributing factors linked to next steps, rather than detailed model transparency. For example, an AI-based CDS tool embedded within the EHR could present a short, structured output (eg, “Increased likelihood of autism based on prior developmental concerns, language delay, and screening results”), paired with suggested next steps such as follow-up screening or referral. This type of explanation aligns with clinician preferences for actionable, case-specific information, as reflected in our findings, and is consistent with prior work emphasizing the importance of interpretable and context-specific outputs in AI-based CDS.^50^ Prior work suggests that explanations are most effective when they are context-specific and aligned with how users interpret decisions.^50^

In addition to model explainability, there may be autism-specific considerations. In current screening practices, the caregiver’s role is active in that they identify—through completion of a screener—developmental concerns in their child.^11^ Because AI-based CDS tools predict autism likelihood without additional input from caregivers, the shift from active caregiver input to model-based identification may influence how results are interpreted and used in clinical care. Importantly, AI-predications also have potential inaccuracies, stemming from biases in clinical practices that are reflected in the EHR data used to train the AI model, as well as selection bias due to limited geographic scope of included data, both of which may impact model performance and reduce generalizability.^51^ These considerations highlight the importance of aligning CDS outputs with clinical judgment and clearly communicating their role in supporting, not replacing, existing screening practices. In practice, this may include explicitly labeling CDS outputs as decision support, providing rationale linked to observable clinical information, and ensuring that outputs are delivered at points in the workflow where they can inform, rather than interrupt, clinical decision-making. In line with current guidelines, even in the context of an AI-based CDS tool, final determinations on autism likelihood and subsequent steps should be informed by clinical judgment, based on key decision points—including those identified in this study.^52^

It is important to recognize several study limitations. First, the study was carried out within an academic health system in a single metropolitan area, limiting the generalizability of the findings to other healthcare systems and community-based practices. Second, the health system in which the study was conducted follows AAP recommendations on universal autism screening at 18- to 24-month well-child visits and digitally administers and scores developmental screening tools. Therefore, findings related to user tasks and technology-user interactions may differ in environments that follow different workflow practices. Lastly, our sample size was limited and may not represent the diversity of perspectives among clinicians and families across different practice settings.

## Conclusion

This study provides a workflow-informed foundation for developing an AI-based CDS tool for early autism detection that can be integrated within routine clinical workflows, by identifying key user tasks, decision points, and technology interactions that shape autism identification during well-child visits and specifying where CDS can be embedded to support care. Clinician and caregiver input revealed barriers and facilitators to current screening approaches and identified design and content preferences for an AI-based CDS tool, emphasizing the need for transparency, actionable guidance, and integration within the clinical workflow, as well as preferences for visual summaries, educational resources, and EHR integration—design strategies to support shared decision-making and follow-up adherence. Future development should prioritize identifying optimal levels of model explainability, refining how information is presented to support clinical interpretation, and determining ways in which the AI-based CDS tool for early autism detection can complement current screening practices to enhance consistency and effectiveness.

## Supplementary Material

ooag145_Supplementary_Data

## Data Availability

The data underlying this article will be shared on reasonable request to the corresponding author, subject to appropriate institutional and ethical approvals.
