# Supplementary material for: Understanding end-user contexts and identifying design preferences of an artificial intelligence-based clinical decision support tool for early autism detection
Source: JAMIA Open. 2026 Jul 23;9(4):ooag145. doi: 10.1093/jamiaopen/ooag145 (PMC13394496; doi:10.1093/jamiaopen/ooag145)
Supplement: ooag145_Supplementary_Data [file ooag145_supplementary_data.zip › Caregiver_Interview.docx]

Caregiver Interview

Record ID

*Page 1*

Date of Observation:

Site:

Observer:

This is the interview portion of the contextual inquiry. Open-ended questions specific to the user should be documented in the "Notes" column. Show relevant graphics to the providers when indicated.

Caregiver Orientation Script: The purpose of this research study is to develop an approach that supports healthcare providers in early autism screening. This approach is part of a new system that will identify children with a high likelihood of autism and uses existing child electronic health records, an interactive screening app, and a developmental screener called the M-CHAT-R/F. The goal is to design an easy-to-use approach that can be used by your healthcare provider during well-child visits.

We are interested in hearing about your thoughts on the new autism screening and feedback approach being developed by our team.

How do you prepare for well-child visits?

**Preparing for well-child visits**

Do you typically complete paperwork or developmental questionnaires before your 18-24 month well-child visit?

For example, do you complete a COVID symptom-screening questionnaire?


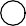
 Yes
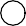
 No

If a concern about your child's development is identified by screening, what kind of information would be helpful for you?

**Right Information**

If your child has a developmental concern after the screening, what would be helpful for you to understand what to do next to help your child (e.g., referrals

and linking to services)?

*Page 2*

**Engagement**

We are designing a new autism screening and feedback approach to identify children with a high likelihood of autism. This approach would include a computer algorithm that uses your child's electronic health history to identify whether your child is at high likelihood of autism and presents this information to your healthcare provider.

Would you have any concerns about this type of screening and feedback approach?


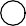
 Yes
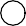
 Maybe
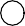
 No

Please list your possible concerns:

Contextual Inquiry Glossary:

Sense to Know App: 6-minute developmental screening app that includes fun videos you watch with your child that gives information on your child's development

Child Developmental Services Agency: an early intervention center for infants and toddlers with developmental disabilities or delay

CDS Presentation: In line with the Five Rights of CDSS, the format or delivery of the clinical decision support (e.g order set, info button, BPA)

CDS Content: In line with the Five Rights of CDS, the information presented by the CDS tool from a reputable and evidence-based source.

Interruptive alert: an alert in electronic health records that requires the provider to take action before moving on Non-interruptive alert: an alert in the electronic health record that does not interfere with the workflow

Acknowledgment Reasons: In order to override an alert, users can select from a coded reason list or provide free-text reasons.

Problem List: used to facilitate continuity of patient care by providing a comprehensive and accessible list of patient problems in one place including illnesses, injury, and other factors that affect an individual's health. The problem list identifies the time of occurrence, identification, and resolution.

Order set/SmartSet: A collection of orders or steps that are aggregated in a single location and used for a given condition, process, or clinical situation (e.g. These can be used within computerized provider order entry systems).

SMART phrase: text that is inserted into a clinical EHR note as a shortcut

*EHR: Electronic Health Record

*CDS: Clinical decision support

*SWYC: Survey of Wellbeing of Young Child

*CDSA: Child Developmental Services Agency

*BPA: Best Practice Advisory

Notes:
