# Supplementary material for: Understanding end-user contexts and identifying design preferences of an artificial intelligence-based clinical decision support tool for early autism detection
Source: JAMIA Open. 2026 Jul 23;9(4):ooag145. doi: 10.1093/jamiaopen/ooag145 (PMC13394496; doi:10.1093/jamiaopen/ooag145)
Supplement: ooag145_Supplementary_Data [file ooag145_supplementary_data.zip › Clinician_Observation_Interview.docx]

Clinician Observation/

Interview

Record ID*Page 1*

Date of Observation:

Site:

Observer:

Use the survey to track specific user workflow from patient check-in to check-out. Keep track of time when indicated including the total length of time the patient is in the exam room and time to score developmental screeners.

Answer each question specific to the user and record details in the "Notes" column.

This is a semi-structured template for observing providers. Observers can document in the "Other" category additional users, tasks, or task characteristics as needed if current options do not apply.

Providers Orientation Script:

The purpose of this research study is to develop a point-of-care clinical decision support system, which aids healthcare providers in early screening for autism and provides referral guidance.. The clinical decision support system will be based on an EHR algorithm that aims to predict the likelihood of autism and incorporates the use of the Sense to Know App, an interactive developmental app, and the M-CHAT-R/F.

We will be conducting observations and interviews with providers from different Primary Care clinics at Duke. The goal is to design a helpful and user-friendly clinical decision support system that is easily incorporated into the workflow of a well-child visit. These observations and interviews will be used to inform the initial prototype design, identify key points for implementation, and to understand requirements you might have.

This will occur in a two-step process: For the first part, I will be observing your activities and tasks during a typical 18-24-month well-child visit.

For the second part, I will be conducting a brief interview to discuss barriers and facilitators when administering autism or other developmental screening tools and when referring patients for services.

Do you have any questions before we begin the observation?

Observation of:
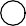
 Nurse
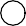
 PCP

Nurse/PCP

**Preparing for well-child visit**

When does the [nurse_pcp] first look at the child's EHR for the well-child visit?

How does the [nurse_pcp] prepare for the well-child visit?

Caregiver

Did the caregiver complete any developmental screeners prior to the well-child visit?


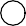
 Yes
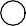
 No

If yes, indicate which developmental screeners were
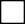
 MCHAT-R

completed prior to the well-child visit?
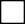
 Ages and Stages
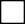
 SWYC


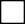
 Other Developmental Screener

Notes:

M-CHAT-R (Preparing for well-child visit)

How was the M-CHAT-R administered to caregivers? Paper Electronic


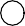

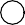

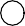

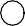


MyChart (prior to well-child visit) Other

Specify Other:

Notes:

Ages and Stages (Preparing for well-child visit)

How is the Ages and Stages Questionnaire administered Paper


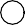

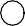

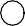

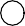


to caregivers? Electronic

MyChart (prior to well-child visit) Other

Specify Other:

Notes:

SWYC (Preparing for well-child visit)

How is the SWYC administered to caregivers? Paper
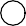
 Electronic Other


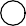

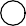


Specify Other:

Notes:

Other Developmental Screener (Preparing for well-child visit)

What is the other developmental screener?

Who gives the other developmental screener to Nurse


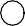

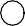

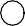

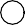

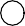


caregivers? Clinic Staff

PCP

MyChart (prior to well-child visit) Other

Specify Other:

How is the other developmental screener administered? Paper
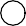
 Electronic


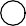

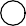


Other

Specify Other:

Notes:

Technical Environment (Preparing for well-child visit)

Nurse/PCP

What tools in the EHR assist the [nurse_pcp] when
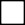
 Note template

preparing for well-child visits? (e.g. health
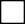
 Order set/SmartSet

maintenance reminder, BPA)
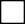
 Best Practice Advisory
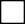
 Other

Specify Other:

Describe the tool and purpose:

General Observation

What other non-developmental clinical decision support
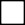
 Note template tools assist while preparing for well-child visits?
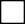
 Order set/SmartSet

(e.g. health maintenance screen, BPA)
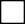
 Best Practice Advisory
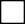
 Other

Specify Other:

Describe the tool and purpose:

Notes:

Caregiver

**Pre-rooming activities (waiting room)**

Does the caregiver complete developmental screeners in the waiting room?


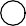
 Yes
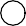
 No

If yes, indicate which developmental screeners were
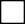
 MCHAT-R

completed in the waiting room?
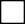
 Ages and Stages
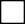
 SWYC


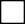
 Other Developmental Screener

Notes:

M-CHAT-R (Pre-rooming activities (waiting room))

Who gives the M-CHAT-R to the caregiver? Nurse Clinic Staff PCP Other


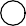

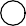

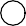

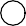


Specify Other:

How was the M-CHAT-R administered to caregivers? Paper Electronic


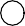

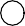

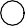

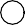


MyChart (prior to well-child visit) Other

Specify Other:

How is the M-CHAT-R scored? Electronic (e.g. tablet or EHR) Microsoft Excel program Paper ( e.g. Scoring template) Other


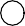

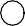

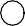

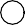


Specify Other:

If scored done by hand, who scores the M-CHAT-R? Nurse Clinic Staff


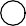

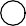

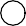

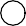


PCP Other

Specify Other:

How long does it take to score the M-CHAT-R?

Notes:

Ages and Stages (Pre-rooming activities (waiting room))

Who gives the Ages and Stages Questionnaire to the caregiver?

Nurse Clinic Staff

PCP Other


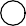

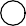

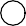

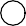


Specify Other:

How is the Ages and Stages Questionnaire administered to caregivers?

Paper
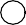
 Electronic Other

Specify Other:


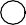

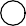


Notes:

| SWYC (Pre-rooming activities (waiting room)) |  | | |
| --- | --- | --- | --- |
| Who gives the SWYC to the caregiver? | Nurse PCP 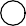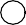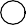 | 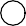 Clinic Staff Other |  |
| Specify Other: |  |  |  |
|  |  |  |  |
| How is the SWYC administered to caregivers? | Paper Other 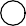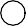 | 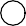 Electronic |  |
| Specify Other: |  |  |  |
|  |  |  |  |
| Notes: |  |  |  |

Other Developmental Screener (Pre-rooming activities (waiting room))

What is the other developmental screener?

Who gives the other developmental screener to caregivers?

Nurse Clinic Staff

PCP Other


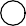

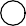

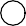

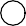


Specify Other:

How is the other developmental screener administered? Paper
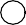
 Electronic


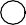

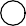


Other

Specify Other:

Notes:

Technical Environment (Pre-rooming activities (waiting room))

Nurse/PCP

What tools in the EHR assist the [nurse_pcp] during
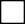
 Note template

pre-rooming activities related to developmental
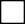
 Order set/SmartSet

screenings (e.g. order sets/ Smart Set, note
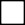
 Best Practice Advisory

templates, BPA)?
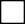
 Other

Specify Other:

Describe the tool and purpose:

General

What other tools are present during pre-rooming
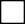
 Note template

activities not related to developmental screenings
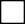
 Order set/SmartSet

(e.g. order sets, note templates, Smart Set, BPA)?
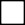
 Best Practice Advisory
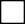
 Other

Specify Other:

Describe the tool and purpose:

Notes:

General Observations

Does the caregiver complete developmental screeners in the patient room?


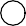
 Yes
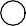
 No

If yes, indicate which developmental screeners were
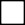
 MCHAT-R

completed in the patient room?
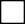
 Ages and Stages
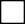
 SWYC


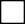
 Other Developmental Screener

Notes:

M-CHAT-R (Administering Screeners)

Who gives the M-CHAT-R to the caregiver? Nurse Clinic Staff PCP


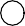

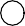

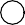

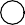

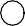


Completed in MyChart (prior to well-child visit) Other

Specify Other:

How is the M-CHAT-R administered to caregivers? Paper Electronic


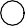

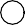

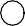


Completed in MyChart (prior to well-child visit) Other

Specify Other:

If completed in a paper format, who records the M-CHAT-R results in the EHR flowsheet?

Nurse Clinic Staff

PCP Other

Specify Other:

How is the M-CHAT-R scored? Electronic (e.g. tablet or EHR) Microsoft Excel program Paper ( e.g. Scoring template) Other

Specify Other:

If scored done by hand, who scores the M-CHAT-R? Nurse Clinic Staff

PCP Other

Specify Other:

How long does it take to score the M-CHAT-R?

Notes:

| Was the MCHAT-R positive? | Yes | No |
| --- | --- | --- |
| For Positive M-CHAT-R Only (Administering Screeners): |  |  |
| Who gives the M-CHAT-R/F to the caregiver? | Nurse PCP | Clinic Staff Other |
| Specify Other: |  |  |

How is the M-CHAT-R/F administered to caregivers? Paper Electronic

Completed in MyChart (prior to well-child visit) Other

Specify Other:

If completed in a paper format, who records the M-CHAT-R/F results in the EHR flowsheet?

Nurse Clinic Staff

PCP Other

Specify Other:

How is the M-CHAT-R/F scored? Electronic (e.g. tablet or EHR) Microsoft Excel program Paper ( e.g. Scoring template) Other

Specify Other:

If done by hand, who scores the M-CHAT-R/F results? Nurse Clinic Staff

PCP Other

Specify Other:

How long does it take to score the M-CHAT-R/F?

Notes:

| Ages and Stages (Administering Screeners) |  | | |
| --- | --- | --- | --- |
| Who gives the Ages and Stages Questionnaire to the caregiver? | Nurse PCP | Clinic Staff Other |  |
| Specify Other |  |  |  |
|  |  |  |  |
| How is the Ages and Stages Questionnaire administered to caregivers? | Paper Other | Electronic |  |
| Specify Other: |  |  |  |
|  |  |  |  |
| If completed in a paper format, who records the Ages and Stages results in the EHR flowsheet? | Nurse PCP | Clinic Staff Other |  |
| Specify Other: |  |  |  |

How is the Ages and Stages Questionnaire scored? Electronic (e.g. tablet or EHR)

Microsoft Excel program Paper ( e.g. Scoring template) Other

Specify Other:

If done by hand, who scores the Ages and Stages Nurse

results? Clinic Staff

PCP

Completed in MyChart (prior to well-child visit) Other

Specify Other:

How long does it take to score the Ages and Stages Questionnaire?

Notes:

| SWYC (Administering Screeners) |  |  |  |
| --- | --- | --- | --- |
| Who gives the SWYC to the caregiver? | Nurse PCP | Clinic Staff Other |  |
| Specify Other: |  |  |  |
|  |  |  |  |
| How is the SWYC administered to caregivers? | Paper Other | Electronic |  |
| Specify Other: |  |  |  |
|  |  |  |  |
| If completed in a paper format, who records the SWYC results in the EHR flowsheet? | Nurse PCP | Clinic Staff Other |  |
| Specify Other: |  |  |  |

How is the SWYC scored? Electronic (e.g. tablet or EHR) Microsoft Excel program Paper ( e.g. Scoring template) Other

Specify Other:

If done by hand, who scores the SWYC results? Nurse Clinic Staff PCP

Completed in MyChart (prior to well-child visit) Other

Specify Other:

How long does it take to score the SWYC?

Notes:

Other Developmental Screener (Administering Screeners)

What is the other developmental screener?

Who gives the other developmental screener to caregivers?

Nurse Clinic Staff

PCP Other

Specify Other:

How is the other developmental screener administered? Paper Electronic

Other

Specify Other:

If completed in a paper format, who records the other developmental screener results in the EHR flowsheet?

Nurse Clinic Staff

PCP Other

Specify Other:

How is the other developmental screener scored? Electronic (e.g. tablet or EHR)

Microsoft Excel program Paper ( e.g. Scoring template) Other

Specify Other:

If done by hand, who scores the other developmental Nurse

screener results? Clinic Staff

PCP

Completed in MyChart (prior to well-child visit) Other

Specify Other:

| How long does it take to score the other developmental screener? |  |  |  | |
| --- | --- | --- | --- | --- |
|  |  |  |  |  |
| Nurse |  |  |  |  |
| Does the nurse interact with the developmental screening results? | Yes No |  |  |  |
| How? |  |  |  |  |
|  |  |  |  |  |
| PCP - M-CHAT-R |  |  |  |  |
| Where does the PCP first look at M-CHAT-R results? | Exam Room Other | Office |  |  |
| Specify Other: |  |  |  |  |
|  |  |  |  |  |
| How are the M-CHAT-R results displayed for the PCP? | EHR Paper | Other |  |  |
| Specify Other: |  |  |  |  |
|  |  |  |  |  |
| When in the workflow does the PCP look at the M-CHAT-R results? |  |  |  |  |
|  |  |  |  |  |
| PCP - For positive M-CHAT-R only: |  |  |  |  |
| Where does the PCP first look at M-CHAT-R results? | Exam Room Other | Office |  |  |
| Specify Other: |  |  |  |  |
|  |  |  |  |  |
| How are the M-CHAT-R results displayed for the PCP? | EHR Paper | Other |  |  |
| Specify Other: |  |  |  |  |
|  |  |  |  |  |
| When in the workflow does the PCP look at the M-CHAT-R results? |  |  |  |  |
|  |  |  |  |  |
| PCP - Ages and Stages and SWYC |  |  |  |  |
| Where does the PCP first look at Ages and Stages Questionnaire results? | Exam Room Other | Office |  |  |
| Specify Other: |  |  |  |  |
|  |  |  |  |  |
| When in the workflow does the PCP look at the Ages and Stages Questionnaire results? |  |  |  |  |

| Where does the PCP first look at the SWYC results? | Exam Other | Room | Office |  | |
| --- | --- | --- | --- | --- | --- |
| Specify Other: |  |  |  |  |  |
|  |  |  |  |  |  |
| When in the workflow does the PCP look at the SWYC results? |  |  |  |  |  |
|  |  |  |  |  |  |
| How are the Ages and Stages Questionnaire results displayed for the PCP? | EHR | Paper | Other |  |  |
| Specify Other: |  |  |  |  |  |
|  |  |  |  |  |  |
| How are the SWYC results displayed for the PCP? | EHR | Paper | Other |  |  |
| Specify Other: |  |  |  |  |  |
|  |  |  |  |  |  |
| PCP - Other Developmental Screener |  |  |  |  |  |
| What is the other developmental screener? |  |  |  |  |  |
|  |  |  |  |  |  |
| Where does the PCP first look at the other developmental screener results? | Exam Other | Room | Office |  |  |
| Specify Other: |  |  |  |  |  |
|  |  |  |  |  |  |
| When in the workflow does the PCP look at the other developmental screening results? |  |  |  |  |  |
|  |  |  |  |  |  |
| How are the Other Developmental Screener results displayed for the PCP? | EHR | Paper | Other |  |  |

Specify Other:

Technical Environment (Administering Screeners)

Nurse/PCP

What tools in the EHR assist the [nurse_pcp] during Note template

the administration of developmental screeners (e.g. Order set/SmartSet order sets/ Smart Set, note templates, BPA)? Best Practice Advisory

Other

Specify Other:

Describe the tool and purpose:

General

What are the other (if any) non-developmental clinical Note template decision support tools used during the well child Order set/SmartSet

visit (e.g. order sets/ Smart Set, note templates, Best Practice Advisory

BPA)? Other

Specify Other:

Describe the tool and purpose:

Notes:

PCP

**Feedback on results / Conclusions**

Does the PCP go over the screening results of the M-CHAT-R with the caregiver?

Yes No

What means of communication (in person, phone call,

MyChart message, etc.) does the PCP use to relay the results of the M-CHAT-R to the caregiver?

If positive M-CHAT-R:

Does the PCP go over the results of the M-CHAT-R/F with the caregiver?

Yes No

What means of communication (in person, phone call,

MyChart message, etc.) does the PCP use to relay the results of the M-CHAT-R to the caregiver?

Does the PCP go over the results of Ages and Stages with the caregiver?

Yes No

What means of communication (in person, phone call,

MyChart message, etc.) does the PCP use to relay the results of the Ages and Stages to the caregiver?

Does the PCP go over the results of SWYC with the caregiver?

Yes No

What means of communication (in person, phone call,

MyChart message, etc.) does the PCP use to relay the results of the SWYC to the caregiver?

Does the PCP go over the results of any other screeners with the caregiver?

Yes No

Which screeners?

What means of communication (in person, phone call, MyChart message, etc.) does the PCP use to relay the results of the other screeners?

What actions does the PCP take, and what resources and Referral to CDSA

support does the PCP provide after a positive Referral for further evaluations within Duke or M-CHAT-R, M-CHAT-R/F, or developmental concern (mark the community [not CDSA]

all that apply)? Referral for intervention services within Duke or the community [not CDSA]

Monitoring [wait and see] No action

Other

Specify Other:

Nurse/PCP

What tools in the EHR assist the [nurse_pcp] during Note template

the feedback stage (e.g. order sets/ Smart Set, note Order set/SmartSet templates, BPA)? Best Practice Advisory

Other

Specify Other:

Describe the tool and purpose:

What tools in the EHR assist the [nurse_pcp] when Note template

concluding well-child visits (e.g. order sets/ Smart Order set/SmartSet

Set, note templates, BPA)? Best Practice Advisory Other

Specify Other:

Describe the tool and purpose:

General

What are the other non-developmental clinical decision Note template support tools present during the feedback stage (e.g. Order set/SmartSet order sets/ Smart Set, note templates, BPA)? Best Practice Advisory

Other

Specify Other:

Describe the tool and purpose:

What other tools are present when concluding Note template

well-child visits not relating to developmental Order set/SmartSet screening (e.g. order sets/ Smart Set, note templates, Best Practice Advisory BPA)? Other

Specify Other:

Describe the tool and purpose:

Notes:

Is the Interview portion applicable? Yes No

**Assessing Context**

This is the interview portion of the contextual inquiry. We are designing a new system for identifying children with a high likelihood of autism. This would include an EMR algorithm that uses claims data to predict the likelihood of autism, a developmental screening app called Sense to Know, and the use of the M-CHAT-R/F. The system would then provide recommendations on the next steps. I will be showing different graphics that will help inform the system we are designing.

Open-ended questions specific to the user should be documented in the "Notes" column. Show relevant graphics to the providers when indicated.

Nurse

What is helpful for you when administering developmental screeners?

What challenges do you face when administering developmental screeners?

Contextual Inquiry Glossary:

Sense to Know App: 6-minute developmental screening app that includes fun videos you watch with your child that gives information on your child's development

Child Developmental Services Agency: an early intervention center for infants and toddlers with developmental disabilities or delay

CDS Presentation: In line with the Five Rights of CDSS, the format or delivery of the clinical decision support (e.g order set, info button, BPA)

CDS Content: In line with the Five Rights of CDS, the information presented by the CDS tool is from a reputable and evidence-based source.

Interruptive alert: an alert in electronic health records that requires the provider to take action before moving on

Non-interruptive alert: an alert in the electronic health record that does not interfere with the workflow Acknowledgment Reasons: In order to override an alert, users can select from a coded reason list or provide free-text reasons.

Problem List: used to facilitate continuity of patient care by providing a comprehensive and accessible list of patient problems in one place including illnesses, injuries, and other factors that affect an individual's health. The problem list identifies the time of occurrence, identification, and resolution.

Order set/SmartSet: A collection of orders or steps that are aggregated in a single location and used for a given condition, process, or clinical situation (e.g. These can be used within computerized provider order entry systems).

SMART phrase: text that is inserted into a clinical EHR note as a shortcut

*EHR: Electronic Health Record

*CDS: Clinical decision support

* SWYC: Survey of Wellbeing of Young Child

*CDSA: Child Developmental Services Agency

*BPA: Best Practice Advisory

PCP

When during a well-child visit do you typically think that a child may be autistic?

What factors into your decision to refer a child for further evaluation or services? (e.g. parent/caregiver concern, provider concern, and/or developmental screening results)

What is helpful for you in deciding whether or not a child might need an autism evaluation?

How do you determine a plan for the next steps for your patients after parents identify autism-related behaviors?

What challenges do you face when administering autism or other developmental screening tools?

What challenges do you face when referring children for services?

What is helpful for you when administering autism or other developmental screening tools?

What is helpful for you when referring children for services?

How does the EHR support you or not support you when screening for autism (e.g. alerts, note templates,

etc.)?

How does the EHR support you or not support you when referring patients (e.g. alerts, note templates,

etc.)?

**Five Rights of Clinical Decision Support**

**Right Information: Information that is conveyed through the CDS Right Format: The presentation of the CDS**

**Right Channel: The platform that delivers the CDS**

**Right Time: The appropriate time in the workflow for the CDS to be deployed Right Person: The right audience who can take action**

Graphic 1. Duke Sense to Know App BPA Alert

Observer Script: Duke Sense to Know Study is validating a developmental screening app. In order to recruit participants, the study team developed a BPA Alert that was incorporated into many Duke Primary Care Clinics. BPA Alerts, best practice advisories, are tools that remind and guide users through clinical activities.

There are two sections of the S2K BPA alert to complete.

The Documentation section includes Medical History Risk Factors, Red Flags, and S2K Study Information.

The Acknowledgement Reasons section is used to override an alert. Users can select from a coded reason list ("Remind me in 1 hr" or "No risks identified")

1. What are your thoughts on this BPA alert (does it seem useful? Does it seem complex or easy to navigate? Is it intuitive?)?

Would you have any difficulties engaging with the alert?

What are your thoughts on the acknowledgment reason options?

The clinical decision support system we are designing will assist in identifying children with a high likelihood of autism.

1. Would you like this alert to be intrusive or non-intrusive?

Intrusive Non-intrusive

An intrusive alert could flag patients at the point of care and provide recommendations on the next steps. A non-intrusive alert could provide you with a list of children based on their likelihood.

Graphic 2: Child Health and Development Interactive System: Asthma Module

Observer:

The Pediatric Asthma Control and Communication Instrument (PACCI) is a validated tool used to measure asthma control. PACCI provides a control score that graphically represents severity over time through a Patient Specific Template.

These are the different features of the alert:

Score Report: The line graph displays the PACCI control score {Lower score indicates a better control similar to the M-CHAT-R}.

Control: The bar chart displays the level of control, control adherence, and interventions used.

Graphic 2 displays the patient's asthma control score using a bar and line graph.

Would you like to view autism screening results in a similar format?

Higher scores could mean a higher likelihood of autism.

Yes No

We are designing a new system for identifying children with a high likelihood of autism. This would include an EMR algorithm that uses claims data to predict the likelihood of autism, a developmental screening app called Sense to Know, and the use of the M-CHAT-R/F.

What format do you prefer to view each of these screening results?

Would you like a summary of the results or a breakdown of each screening result?

The new system we are designing will screen for autism using the following: the prediction model, the Sense to Know App, and the M-CHAT-R/F. Based on the results, the system will then provide PCPs with recommendations on the next steps (i.e. referrals, community support, therapies, etc.).

Would this be helpful for you when screening for autism?

Yes No

Graphic 3. Likelihood Stratification

Observer Script: This is a two-step algorithm that identifies and sorts patients into three groups based on the likelihood of conditions. Clinics utilize this likelihood stratification to assist in the appropriate management of care.

Features:

Low, Medium, and High Likelihood Stratification

The system we are developing will be identifying patients who are at a low, medium, and high likelihood of autism.

Based on this stratification, how would you like this information presented to you? Graphic 3.

When would you like to have this information presented to you?

Would you like to have recommendations on the next steps based on likelihood and how to implement them?

Would you like to have a script that explains how the algorithm works and the next steps for caregivers?

Contextual Inquiry Glossary:

Sense to Know App: 6-minute developmental screening app that includes fun videos you watch with your child that gives information on your child's development

Child Developmental Services Agency: an early intervention center for infants and toddlers with developmental disabilities or delay

CDS Presentation: In line with the Five Rights of CDSS, the format or delivery of the clinical decision support (e.g order set, info button, BPA)

CDS Content: In line with the Five Rights of CDS, the information presented by the CDS tool from a reputable and evidence-based source.

Interruptive alert: an alert in electronic health records that requires the provider to take action before moving on Non-interruptive alert: an alert in the electronic health record that does not interfere with the workflow

Acknowledgement Reasons: In order to override an alert, users can select from a coded reason list or provide free-text reasons.

Problem List: used to facilitate continuity of patient care by providing a comprehensive and accessible list of patient problems in one place including illnesses, injury, and other factors that affect an individual's health. The problem list identifies the time of occurrence, identification, and resolution.

Order set/SmartSet: A collection of orders or steps that are aggregated in a single location and used for a given condition, process, or clinical situation (e.g. These can be used within computerized provider order entry systems). SMART phrase: text that is inserted into a clinical EHR note as a shortcut

*EHR: Electronic Health Record

*CDS: Clinical decision support

*SWYC: Survey of Wellbeing of Young Child

*CDSA: Child Developmental Services Agency

*BPA: Best Practice Advisory
